# Multi-level transcriptome sequencing identifies *COL1A1* as a candidate marker in human heart failure progression

**DOI:** 10.1186/s12916-019-1469-4

**Published:** 2020-01-06

**Authors:** Xiumeng Hua, Yin-Ying Wang, Peilin Jia, Qing Xiong, Yiqing Hu, Yuan Chang, Songqing Lai, Yong Xu, Zhongming Zhao, Jiangping Song

**Affiliations:** 1 Department of Cardiac Surgery, State Key Laboratory of Cardiovascular Disease, Fuwai Hospital, National Center for Cardiovascular Diseases, Chinese Academy of Medical Sciences and Peking Union Medical College, 167A Beilishi Road, Xi Cheng District, Beijing, 10037 China; 2 Center for Precision Health, School of Biomedical Informatics, The University of Texas Health Science Center at Houston, 7000 Fannin St, Houston, TX 77030 USA; 3 Children’s Nutrition Research Center, Department of Pediatrics, Baylor College of Medicine, One Baylor Plaza, Houston, TX 77030 USA; 4 Human Genetics Center, School of Public Health, The University of Texas Health Science Center at Houston, 1200 Pressler St, Houston, TX 77030 USA; 5 Department of Biomedical Informatics, Vanderbilt University Medical Center, 2525 West End Avenue, Nashville, TN 37203 USA

**Keywords:** Heart failure, Transcriptomics, microRNA, lncRNA, Regulatory network, Fibrosis

## Abstract

**Background:**

Heart failure (HF) has been recognized as a global pandemic with a high rate of hospitalization, morbidity, and mortality. Although numerous advances have been made, its representative molecular signatures remain largely unknown, especially the role of genes in HF progression. The aim of the present prospective follow-up study was to reveal potential biomarkers associated with the progression of heart failure.

**Methods:**

We generated multi-level transcriptomic data from a cohort of left ventricular heart tissue collected from 21 HF patients and 9 healthy donors. By using Masson staining to calculate the fibrosis percentage for each sample, we applied lasso regression model to identify the genes associated with fibrosis as well as progression. The genes were further validated by immunohistochemistry (IHC) staining in the same cohort and qRT-PCR using another independent cohort (20 HF and 9 healthy donors). Enzyme-linked immunosorbent assay (ELISA) was used to measure the plasma level in a validation cohort (139 HF patients) for predicting HF progression.

**Results:**

Based on the multi-level transcriptomic data, we examined differentially expressed genes [mRNAs, microRNAs, and long non-coding RNAs (lncRNAs)] in the study cohort. The follow-up functional annotation and regulatory network analyses revealed their potential roles in regulating extracellular matrix. We further identified several genes that were associated with fibrosis. By using the survival time before transplantation, *COL1A1* was identified as a potential biomarker for HF progression and its upregulation was confirmed by both IHC and qRT-PCR. Furthermore, COL1A1 content ≥ 256.5 ng/ml in plasma was found to be associated with poor survival within 1 year of heart transplantation from heart failure [hazard ratio (HR) 7.4, 95% confidence interval (CI) 3.5 to 15.8, Log-rank *p* value < 1.0 × 10^− 4^].

**Conclusions:**

Our results suggested that COL1A1 might be a plasma biomarker of HF and associated with HF progression, especially to predict the 1-year survival from HF onset to transplantation.

## Background

Heart failure (HF), a chronic condition characterized by structural and functional impairment of the heart, has been recognized as a global pandemic and is increasing in prevalence [1–3]. During the past decades, the diagnosis of HF has been mainly based on echocardiography including dilation of left ventricular (LV) and cardiac dysfunction (left ventricular ejection fraction, or LVEF, < 40%). Regardless of the etiology, it has shown that the underlying mechanisms contributing to the progression of HF can lead to stereotypical changes in gene expression [4]. Therefore, investigation of transcriptional profiles and related changes may gain new insights into its molecular mechanisms, helping us develop better diagnostic and prognostic strategies.

Cardiac fibrosis is a requisite component that underlies nearly all forms of HF. With the advent of anti-fibrotic pharmacologic therapies, fibrosis has become an important therapeutic target in HF patients [5]. Moreover, fibrosis disrupts the myocardial architecture, thereby predisposing the progression of cardiac diseases to HF [6]. To our knowledge, there have been limited studies reporting the role of specific fibrosis genes in HF progression which can be used as biomarkers in diagnosis and prognosis. Recent advances in transcriptional profiling allow us to not only investigate the mRNA level, but also non-coding RNA including microRNA (miRNA) and long non-coding RNA (lncRNA). Studies have reported that several mRNAs (e.g., *CORIN*, *CTGF*, and *POSTIN*), miRNAs (e.g., miR-1, miR-133 and miR-423-5p), and lnRNAs (e.g., H19 and HOTAIR) might play roles in the pathogenic mechanisms leading to HF [7–11]. Although these studies have reported many promising findings, a systematic investigation of multiple types of expression and their regulation in HF will likely reveal more dynamic and regulatory signatures related to fibrosis in HF, thus helping us better understand the development and progression of HF.

**Fig. 1:**
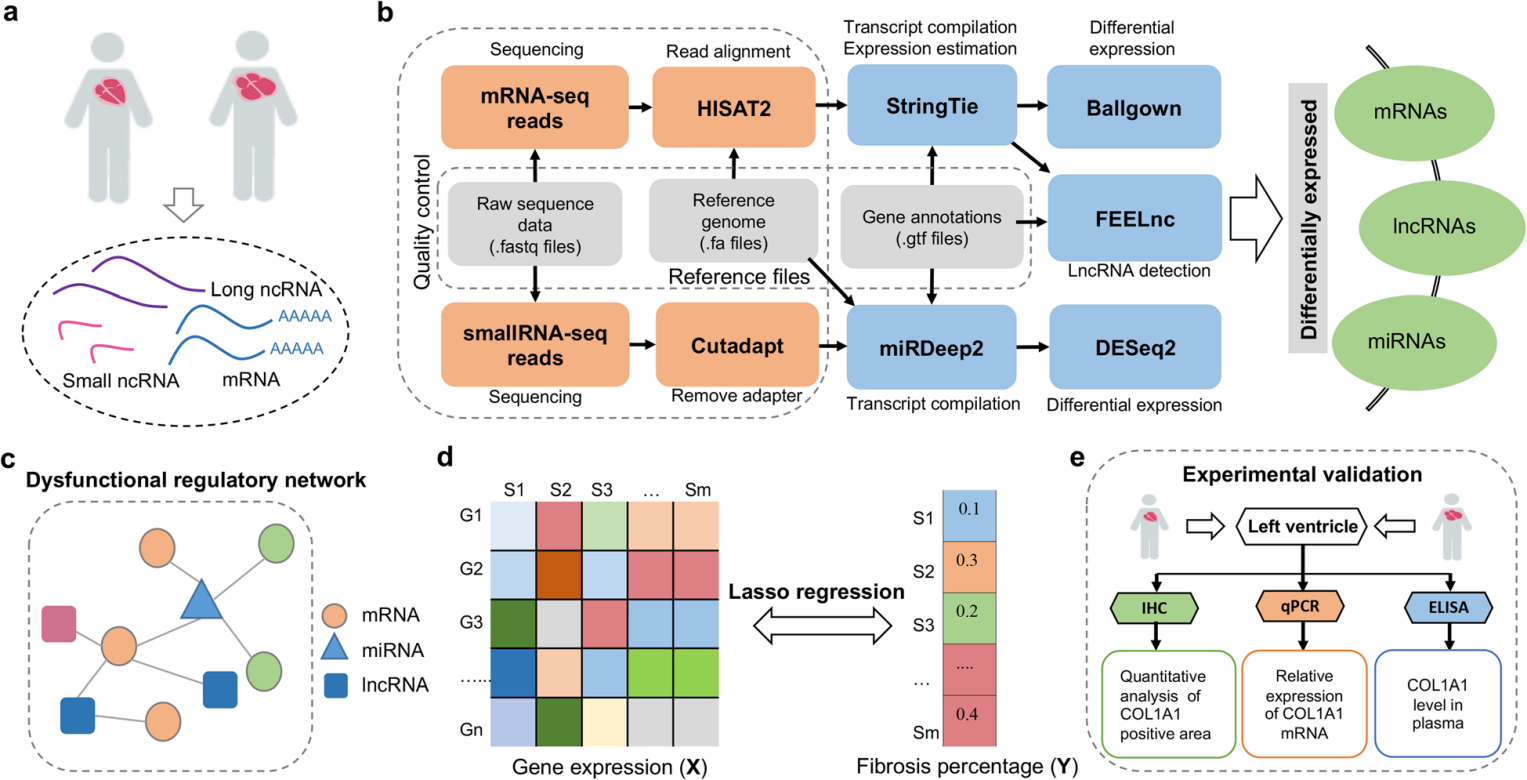
Flowchart for uncovering novel molecular signatures in heart failure using multi-transcriptome approach. **a** Clinical specimen collection and multi-level RNA sequencing. **b** Pipeline for mRNA, lncRNA, and microRNA (miRNA) analysis. **c** Construction of regulatory networks in heart failure using differentially expressed mRNAs, miRNAs, and lncRNAs. **d** Lasso regression analysis of the association between gene expression and fibrosis percentage in heart failure tissue. **e** Experimental validation of *COL1A1* being associated with HF progression

In this study, we collected the left ventricular tissue from 21 HF patients and 9 healthy donors for whole transcriptome sequencing (mRNA and lncRNA) and small RNA sequencing (miRNA). Our functional enrichment analysis of differentially expressed genes (DEGs) revealed significant pathways, including extracellular matrix (ECM) that might play important roles in HF. By constructing the dysfunctional regulatory networks, several miRNAs (e.g., miR-129-5p and miR-190-5p) and lncRNAs (e.g., BANCR and PDZRN3-AS1) were pinpointed to be critical in HF. Using a lasso regression method, several genes, especially ECM-related genes, were identified to contribute to fibrosis, a main feature of HF. In particular, an upregulation of *COL1A1* in HF, which was regulated by miRNA miR-190-5p and lncRNA MSTRG.16534 in our regulatory network, was found to be related to fibrosis. Interestingly, the COL1A1 content in plasma was found to contribute to the progression of HF, suggesting that it might be a potential plasma biomarker to predict the heart transplantation (HTx) within 1 year from HF onset. These findings suggested the possible roles of ECM, in particular via a *COL1A1* regulatory module, in the progression of HF. The flowchart of our study is illustrated in Fig. 1.

## Methods

### Human LV tissue acquisition and RNA extraction

This study complies with the ethical guidelines of the 1975 Declaration of Helsinki. All participants provided written informed consent at the time of enrollment. We recruited 21 heart failure patient hearts from Fuwai Hospital heart transplantation database with left ventricle (LV) tissue obtained at the time of HTx and preserved in liquid nitrogen. The 9 healthy LV heart samples were obtained from brain-death donors with normal circulatory supply, who were not suitable for transplantation due to the technical or non-cardiac reasons such as body-weight mismatch by following the guideline of China Transplant Services. All of patients received standard drug therapy against HF before HTx [12]. Information of demographic characteristics, comorbidities, ultrasonic cardiogram, medication, and arrhythmia history was collected and summarized in Table 1.

**Table 1:** Selected characteristics at presentation in 21 heart failure patients and healthy donors

Basic information	Heart failure patients *n* (percentage of 21)	Healthy donors	*p* value
Age (years)	34.6 ± 15.9	41.7 ± 4.0	0.07
Male (percentage)	13 (61.9)	9(100)	0.07
NYHA functional class, *n* (percentage of 21)			
II	2 (9.5)		
III	7 (33.3)		
IV	12 (57.1)		
Comorbidities			
Diabetes mellitus	2 (9.5)		
Smoking history	5 (23.8)		
Pulmonary hypertension	5 (23.8)		
Tricuspid regurgitation	12 (57.1)		
Mitral regurgitation	17 (81.0)		
Ultrasonic cardiogram			
LVEF (%)	27.6 ± 9.8		
LA (mm)	44.7 ± 4.8		
IVS-thickness (mm)	8.2 ± 1.1		
LVEDD (mm)	65.1 ± 16.6		
Arrhythmia history, *n* (percentage of 21)			
Atrial fibrillation	4 (19.0)		
Premature ventricular contraction	6 (28.6)		
Ventricular tachycardia	4 (19.0)		
Ventricular fibrillation	1 (4.8)		
CRBBB	3 (14.3)		
Drug therapy, *n* (percentage of 21)			
ASA	2 (9.5)		
Amio	4 (19.0)		
β-Blocker	15 (71.4)		
Digoxin	16 (76.2)		
ACEI/ARB	17 (81.0)		
CCB	3 (14.3)		
Diuretic	18 (85.7)		

The total RNA was extracted from the frozen LV samples using the Trizol protocol. RNA concentration was measured using Qubit® RNA Assay Kit in Qubit® 2.0 Flurometer (Life Technologies, CA, USA), and RNA integrity was assessed using the RNA Nano 6000 Assay Kit of the Bioanalyzer 2100 system (Agilent Technologies, CA, USA). Only RNA samples with a total amount of at least 6 μg and RNA integrity number (RIN) of at least 6.9 (range 6.9 to 8.5, mean ± SD 7.9 ± 0.4) were used for subsequent library construction and sequencing (Additional file 1: Table S1).

### RNA library preparation, clustering, and sequencing

A total amount of 3 μg RNA per sample was used as input material for RNA library preparation. First, ribosomal RNA was removed by Epicentre Ribo-zero™ rRNA Removal Kit (Epicentre, USA), and rRNA-free residue was cleaned up by ethanol precipitation. Next, sequencing libraries were generated using the rRNA-depleted RNA by NEBNext® Ultra™ Directional RNA Library Prep Kit for Illumina® (NEB, USA) following the manufacturer’s recommendation. Briefly, fragmentation was carried out using divalent cations under elevated temperature in NEBNext First Strand Synthesis Reaction Buffer (5X). First-strand cDNA was synthesized using random hexamer primer and M-MuLV Reverse Transcriptase (RNaseH^−^). Second-strand cDNA synthesis was subsequently performed using DNA Polymerase I and RNase H. In the reaction buffer, dNTPs with dTTP were replaced by dUTP. Remaining overhangs were converted into blunt ends via exonuclease/polymerase activities. After adenylation of 3′ end of DNA fragments, NEBNext Adaptor with hairpin loop structure was ligated to prepare for hybridization. To select cDNA fragments of preferentially 150~200 bp in length, the library fragments were purified with AMPure XP system (Beckman Coulter, Beverly, USA). Then, 3 μl USER Enzyme (NEB, USA) was used with size-selected, adaptor-ligated cDNA at 37 °C for 15 min followed by 5 min at 95 °C before PCR. PCR was performed with Phusion High-Fidelity DNA polymerase, Universal PCR primers, and Index (X) Primer. Finally, the products were purified (AMPure XP system) and library quality was assessed on the Agilent Bioanalyzer 2100 system. The clustering of the index-coded samples was performed on a cBot Cluster Generation System using TruSeq PE Cluster Kit v3-cBot-HS (Illumia) according to the manufacturer’s instructions. After cluster generation, the libraries were sequenced on an Illumina Hiseq 2500 platform and 150-bp paired-end reads were generated.

### Small RNA library construction, clustering, and sequencing

A total amount of 3 μg total RNA per sample was used as input material for the small RNA library preparation. Sequencing libraries were generated using NEBNext® Multiplex Small RNA Library Prep Set for Illumina® (NEB, USA) following the manufacturer’s recommendation, and index codes were added to attribute sequences to each sample. Briefly, NEB 3′ SR Adaptor was directly and specifically ligated to 3′ end of miRNA, siRNA, and piRNA. After the 3′ ligation reaction, the SR RT Primer hybridized to the excess of 3′ SR Adaptor (that remained free after the 3′ ligation reaction) and transformed the single-stranded DNA adaptor into a double-stranded DNA molecule. This step is important to prevent adaptor-dimer formation. In addition, dsDNAs were not substrates for ligation mediated by T4 RNA Ligase 1 and therefore did not ligate to the 5′ SR Adaptor in the subsequent ligation step. 5′ end adapter was ligated to 5′ end of miRNA, siRNA, and piRNA. Then first-strand cDNA was synthesized using M-MuLV Reverse Transcriptase (RNase H^−^). PCR amplification was performed using LongAmp Taq 2X Master Mix, SR Primer for Illumina and index (X) primer. PCR products were purified on an 8% polyacrylamide gel (100 V, 80 min). DNA fragments corresponding to 140~160 bp (the length of small non-coding RNA plus the 3′ and 5′ adaptors) were recovered and dissolved in 8 μl elution buffer. Finally, library quality was assessed on the Agilent Bioanalyzer 2100 system using DNA High Sensitivity Chips. The clustering of the index-coded samples was performed on a cBot Cluster Generation System using TruSeq SR Cluster Kit v3-cBot-HS (Illumia) according to the manufacturer’s instructions. After cluster generation, the libraries were sequenced on an Illumina Hiseq 2500 platform and 50 bp single-end reads were generated.

### Heart failure validation cohort

To validate whether the COL1A1 content in plasma would be used as a biomarker for HF progression, we recruited 139 samples from an independent heart failure cohort in Fuwai Hospital. These patients were qualified heart failure diagnosis and received HTx. In selection of validation samples, those patients were excluded if they were combined with any of the following conditions: (1) hepatitis virus positive such as HBV and HCV, (2) liver cirrhosis or liver cancer, (3) pulmonary fibrosis or myelofibrosis, (4) malignant tumor, or (5) other system diseases. In addition, all the patients had standard medication treatment without mitral valve modeling. The blood samples of these patients were collected and preserved before HTx.

### Histology, electron microscopy, and Masson analysis

To calculate the fibrosis percentage for each sample, tissue processing, section making, HE staining, and Masson trichrome staining were performed according to the previously published procedures [13]. All of the staining sections were scanned as digital images by a slice scanner to further analysis [14]. We performed the Masson analysis by calculating percentages of tissue components with hue, saturation, and intensity independently by three researchers at least one time per person. In each whole section, five randomly selected fields were evaluated under microscope (× 200) from epicardial to endocardial region per whole slice, with excluding trabecular and scar tissue [15].

Besides, the ultrathin section of transplanted heart tissue was described briefly as follows: both ventricles of transplanted heart tissue were routinely fixed in 2.5% glutaraldehyde in 0.1 M/L phosphate buffer (pH 7.3) and post-fixed in buffered 1% osmium tetroxide. Then, the images were acquired by transmission electron microscope.

### Data pre-processing, de novo assembly, and annotation

The raw sequence reads of mRNA/lncRNA were cleaned by removing the RNA adapters and trimming the low-quality bases (Q < 20). For mRNA-seq, approximately 1.33 billion clean reads were generated after removing the adapter by Cutadapt program (version 1.9). Among all the clean reads, more than 97.60% had the Phred-like quality score at the Q20 level (an error probability of 1%). RNA sequencing tags were only considered when they mapped to the same DNA strand as indicated by GRCh38.p11 annotation using HISAT2 (version 2.1.0) [16]. After assembly, approximately 96.74% of the total clean reads were mapped to the reference transcriptome. The fragments per kilobase of transcript per million mapped reads (FPKM) value of 186,363 transcripts was calculated based on StringTie (version 1.3.4) with default parameters [16]. mRNA with low expression were excluded, defined as those with FPKM less than 1 in more than 80% samples. For lncRNAs, we did not remove the low expressed transcripts, while we identified the potential long non-coding transcripts by filtering out short transcripts (default 200 nt) and removing single exon transcripts based on the FEELnc tool (version 0.11-2) [17]. The remaining transcripts were then used to perform the following analysis.

For miRNAs, mature miRNA and precursor miRNAs of human were obtained from miRBase (Release 22) [18]. The reads were first subjected to adapter removal through the Cutadapt program. Approximately 609.59 million clean reads were obtained after removing the adapters and further pre-processed by miRDeep2 [19]. The known mature miRNA expression profile was generated by using the quantifier module of the miRDeep2 package that gives the read counts for the known miRNAs. Specifically, 479.49 million reads (78.70%) were mapped to mature human miRNAs. For the pre-processing of miRNA data, we removed miRNAs with a missing value in > 10% of the samples. According to the miRNA sequence database miRBase [20] (Release 22), there were 2620 mature human miRNAs. Our analysis resulted in 604 expressed miRNAs in our samples.

### Identification of differentially expressed mRNAs, miRNAs, and lncRNAs

The differentially expressed mRNAs and lncRNAs between the samples with and without HF were detected by Ballgown (version 2.16.0) [16] based on the expression levels obtained from StringTie with threshold of adjusted *p* value less than 0.05. We further required the DEGs or DElncs to have more than twofold changes. Similarly, the DEmiRs were obtained by using DEseq2 (version 1.24.0) [21] R package with Benjamini-Hochberg (BH) [22] adjusted *p* value less than 0.05 and more than twofold changes.

We performed functional enrichment analysis of the DEGs using the online tool WebGestalt (2019 version) [23]. We used all the genes only detected in our study as the reference geneset. The pathways and GO terms with adjusted *p* value < 0.05 were considered being statistically enriched. To better interpret the results, we constructed a DEG GO term associated network by using the plugin module ClueGO [24] in Cytoscape [25] with the default parameters. In this network, a node represents a gene or a term while an edge indicates that a gene belongs to a term. To investigate the functions of the DElncs, we identified their potential targets based on the FEELnc tool with default parameter since classifying lncRNAs with mRNAs could help predict the functions of lncRNAs [17]. For the DEmiRs, we performed microRNA Enrichment Analysis and Annotation (miEAA) with Over Representation Analysis (ORA) [26] to detect the significantly enriched categories. The adjusted *p* value cutoff was set to 0.05.

### Construction of regulatory network for HF

To construct the miRNA-gene regulatory network, the target genes of miRNAs were collected from both computational prediction and experimental validation. Three computational methods were employed to predict the target genes of miRNAs, including PITA [27], miRanda (August 2010 Release) [28], and TargetScan (Release 7.1) [29]. We selected the miRNA and target gene pairs supported by at least two tools to avoid false positives [30]. We further collected experimentally determined miRNA target genes deposited in miRTarBase (Release 7.0) [31]. In total, a set of 502,768 miRNA-gene interactions involving 2600 miRNA and 16,732 genes were obtained. To build the miRNA-DEG regulatory network, we considered only the DEmiRs as they were more likely to be related to HF. Furthermore, we required the miRNAs to be negatively correlated with the target genes (all were DEGs in this analysis) or regulate DEGs through its target genes. The relationship was measured by the Spearman’s correlation coefficient (*p* value< 0.05) in the HF samples.

Similarly, we constructed a lncRNA-gene interaction network by including DElncs and their co-expressed DEGs (*p* value< 0.05 for Spearman’s correlation coefficient). This was built on the assumption that lncRNAs may regulate their target genes, although they may not directly bind to the genes [32].

### Identification of fibrosis-related genes based on lasso regression

To identify the genes that were most associated with fibrosis, we fitted a lasso regression model for feature selection. We used the gene expression as the exposure variable and the fibrosis percentage for each sample as the response variable. By applying to our dataset, it would pick up a group of genes whose expression profile could best explain the fibrosis level in HF patients. Here, the percentage of fibrosis was calculated following the method in Ref. [13]. Among the 21 HF samples, 18 had the data for the percentage of fibrosis.

In this study, we applied the Python package scikit-learn (version 0.20.0) to solve the problem mentioned above.

### Immunohistochemical analysis of COL1A1

Immunohistochemical staining of COL1A1 was performed according to the following protocol. Formalin fixed paraffin-embedded sections of LV were dewaxed by methanol, subjected to antigen retrieval (heat mediation in an EDTA buffer, pH = 9.0), blocked for 30 min, incubated at 4 °C overnight with anti-collagen I antibody at 1:200 dilution (Abcam, ab34710), then incubated at room temperature with a secondary antibody: HRP conjugated rabbit IgG. The whole slice was scanned by a digital scanner, and the COL1A1-positive area within the slice was calculated by Image-Pro Plus Version 6.0 (Media Cybernetics).

### Validation of *COL1A1* expression by quantitative real-time PCR (qRT-PCR)

Total RNA was extracted according to the Trizol protocol while complementary DNA (cDNA) was synthesized by PrimeScript RT Master Mix kit (Takara, RR036A). Each cDNA was diluted by 20 folds and then used as a template for qRT-PCR assay using SYBR Green Master Mix (Thermo Fisher Scientific, A25742). The qRT-PCR was performed in a 10 μl reaction volume by Applied Biosystems®ViiA7 Real-Time Thermo Fishers (Thermo Fisher Scientific, USA). Three technical replicates were assayed for each reaction. The procedure for qRT-PCR was as follows: 30 s at 95 °C for denaturation, followed by 40 cycles at 95 °C for 10 s, 60 °C for 20 s and 72 °C for 20 s. The relative expression value of the selected genes was calculated using the 2^−ΔΔCT^ method in the Applied Biosystems®ViiA7 Real-Time PCR Systems. The primers of *COL1A1* were F: 5′-GATTCCCTGGACCTAAAGGTGC-3′ and R: 5′-AGCCTCTCCATCTTTGCCAGCA-3′. The primers for *GAPDH* were F: 5′-GGAGCGAGATCCCTCCA-3′ and R: 5′-GGCTGTTGTCATACTTCTCATGG-3′.

### Quantitative enzyme-linked immunosorbent assay (ELISA)

Frozen plasma samples that were stored in a − 80 °C freezer were quickly thawed at 37 °C, followed by putting on ice. Human COL1A1 ELISA kit (Abcam, ab210966) was used to analyze plasma samples. All procedures were performed according to the manufacturer’s protocol.

## Results

### Clinical and pathological description of the study cohort

All the enrolled patients in this study were collected from Fuwai Hospital heart transplantation database, including 21 HF patients and 9 healthy donors. The baseline demographic and clinical characteristic of the patients and healthy donors are summarized in Table 1. Patients in our study were diagnosed by clinical and pathological performance: 18 of them were diagnosed as dilated cardiomyopathy (DCM) and the remaining 3 as myocarditis. All patient presenting end-stage HF were treated with standard medication treatment before HTx. The mean ± standard deviation (SD) age was 34.6 ± 15.9. Among them, 61.9% of the patients were men and 90.5% of the patients were with NYHA class III (*n* = 7, 33.3%) or IV (*n* = 12, 57.1%). Most patients (*n* = 19, 90.5%) had reduced LVEF (< 40%), and only 1 patient had HF with a preserved LVEF (≥ 50%). LVEF was not available for one patient (4.8%) and the LVEF ± SD was 27.6 ± 9.8%. Few patients (*n* = 2, 9.5%) had diabetes mellitus history, five patients (23.8%) had smoking history, and some patients (*n* = 5, 23.8%) companied with pulmonary hypertension. Since the ventricle had enlarged, some patients were with different extent of regurgitation of tricuspid or mitral, or both. Six patients (*n* = 6, 28.6%) had premature ventricular contraction and most patients had been treated with diuretic (*n* = 18, 85.7%), ACEI/ARB (*n* = 17, 81.0%), digoxin (*n* = 16, 76.2%), or β-blocker (*n* = 15, 71.4%). All healthy donors were males with the mean ± SD age being 41.7 ± 4.0, which was not significantly different from that of the heart failure patients (41.7 ± 4.0 vs. 34.6 ± 15.9, *t* test *p* value = 0.07). The sex was not significantly different between heart failure patients and healthy donors [13(61.9%) vs. 9(100%), Fisher’s exact test *p* value = 0.07]. In addition, the LVEF of healthy donors was required more than 60% before the donation. The detailed clinical information can be found in Additional file 1: Table S1.

**Fig. 2:**
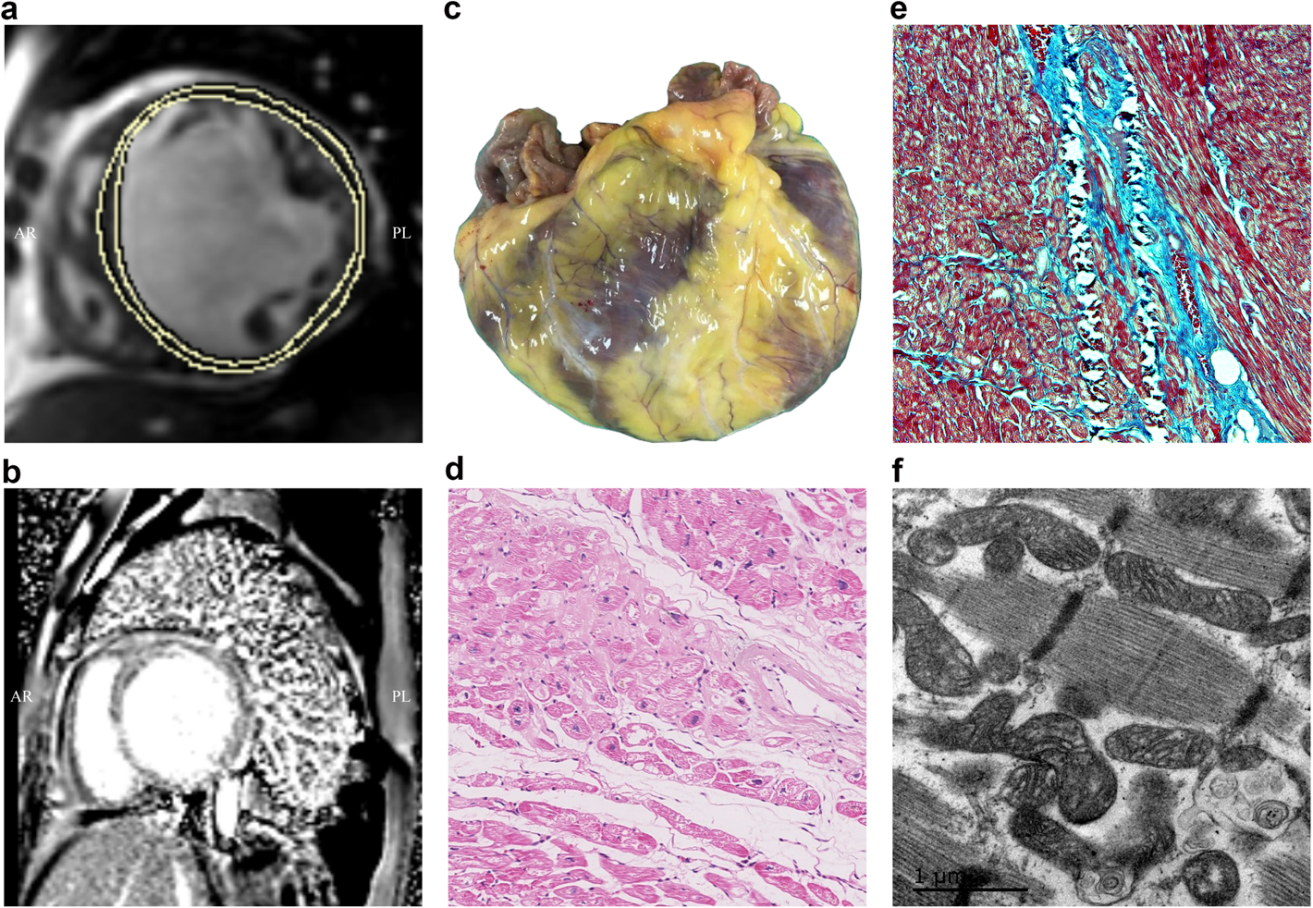
Clinic pathological characteristics of heart failure in this study. **a** cardiac magnetic resonance (CMR) demonstrating short axis T1-weighted imaging of dilated ventricles and dysfunction. **b** Short axis LGE imaging of fibrosis in ventricle walls. **c** Macroscopic performance of the dilated cardiomyopathy (DCM). **d** Different degrees and sarcoplasmic degenerative change of cardiomyocytes in hematoxylin eosin (HE) stain. **e** Fibrosis feature of a heart failure patient. **f** An ultrastructural image of DCM

As shown in Fig. 2a, b, the LV dilatation and dysfunction, evaluated by cardiac magnetic resonance (CMR), were found to occur frequently. In addition, fibrosis in different regions of the LV was found in late gadolinium enhancement (Fig. 2b). Figure 2 c–f show typical pathological characteristics in a HF sample (sample ID: S16). The end-stage HF patients were characteristic with the dilated ventricle cavity (Fig. 2c). Typical features of the heart tissue that underwent HF included different degrees of cardiomyocyte hypertrophy and sarcoplasmic degenerative changes such as the appearance of vacuolization (Fig. 2d). Massive fibrosis was found around the interstitial or perivascular regions (Fig. 2e). The ultrastructure performance also indicated myofilament changes that were apparent in degenerated cardiomyocytes (Fig. 2f).

### The transcriptomic landscape of HF

**Fig. 3:**
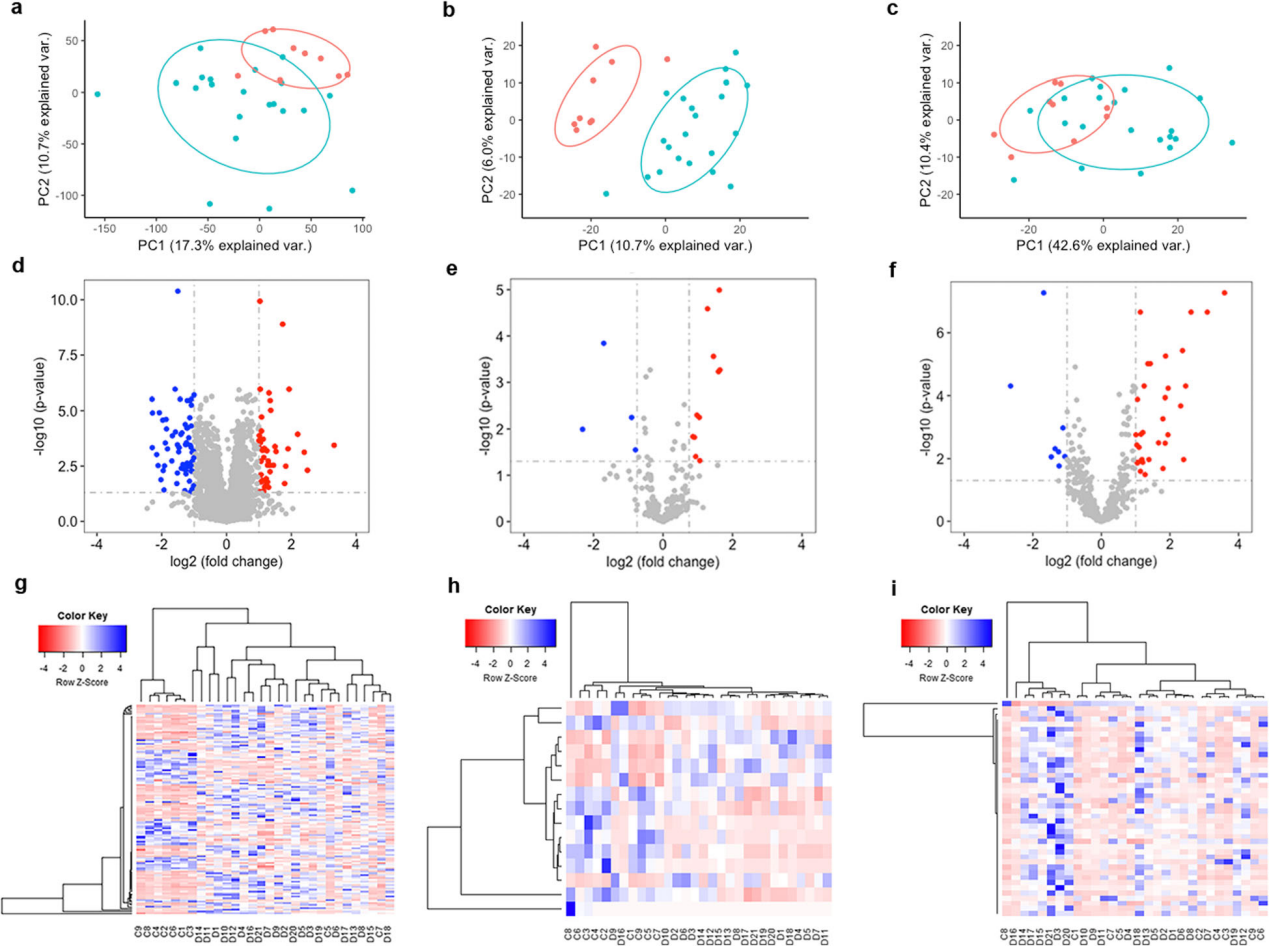
Differential gene expression analysis. **a–c** Principal component analysis (PCA) of mRNAs (**a**), lncRNAs (**b**), and microRNAs (miRNAs) (**c**), respectively. Each dot represents one sample. Blue: heart failure (HF) samples. Red: control samples. **d–f** Volcano plots showing mRNA (**d**), lncRNA (**e**), and miRNA (**f**) differential expression. Red and blue dots denote significantly upregulated and downregulated genes, respectively. **g–i** Heatmap plots showing differentially expressed mRNAs, lncRNAs, and miRNAs among the samples. *X*-axis: sample IDs starting with a D denote HF samples and samples with a C for control samples

RNA-seq data process, quality check, and analysis were described in “Methods”. The detailed sequence statistics, including number of reads, alignment, and mapping information for each sample were summarized in Additional file 2: Table S2. In this study, we refer mRNA to protein-coding gene and lncRNA for long non-coding gene (Table 2). As shown in Fig. 3a–c, principal component analysis (PCA) was first performed for the expression profiles of mRNAs, lncRNAs, and miRNAs. All three RNA types could largely distinguish HF patients from healthy controls. Interestingly, lncRNA expression profiles had the best performance in distinguishing HF from healthy controls, reinforcing the importance to further investigate their underlying mechanism in HF. In Fig. 3d–f, volcano plots showed downregulated (blue nodes) and upregulated (red nodes) genes. We identified 126 mRNAs, 16 lncRNAs, and 42 miRNAs that were differentially expressed in HF versus control samples by requiring the absolute log2-transformed fold change (FC) > 1 and adjusted *p* value < 0.05 by the BH method. Furthermore, unsupervised hierarchical clustering of the expression profiles for the differentially expressed mRNAs revealed a distinct expression signature of HF compared to healthy samples as shown in Fig. 3g–i. It is suggested that HF samples had distinct transcriptomic changes at multiple molecular levels when compared to control samples.

**Table 2:** Summary statistics of the sequencing data

mRNA and lncRNA	Total reads*	Concordantly aligned 0 time	Concordantly aligned once	Concordantly aligned ≥ 2 times	Average alignment rate (%) (mean ± SD)
	1,325,391,991	92,160,909	1,124,273,057	108,958,025	96.74 ± 0.38
miRNA	Total reads*	Mapped	Unmapped	Mapping rate (%) (mean ± SD)	Unmapping rate (%) (mean ± SD)
	609,591,685	479,490,657	130,101,028	78.70 ± 7.01	21.30 ± 7.01

*Data for 21 HF and 9 control samples

**Fig. 4:**
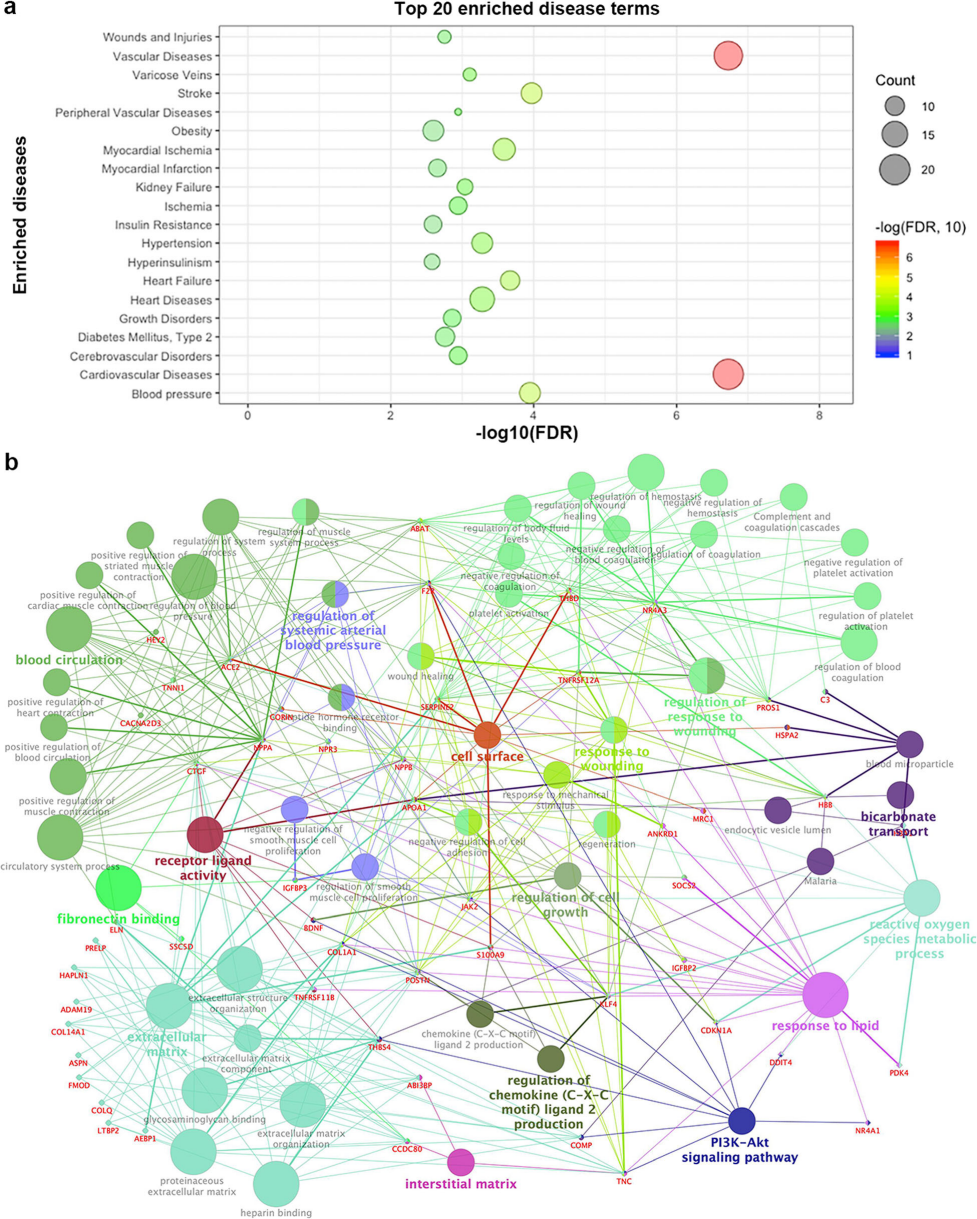
Functional enrichment and network analysis of differentially expressed mRNA genes (DEGs) in heart failure (HF). **a** Top 20 most significantly enriched disease terms. **b** Pathway network for HF DEGs. A circle node denotes the GO BP term. A diamond node denotes a DEG involved in the enriched pathway. Node color denotes different categories

We further performed the functional enrichment analysis of the differentially expressed genes (DEGs). Several well-known diseases related to HF were significantly enriched, such as cardiovascular disease (BH adjusted *p* value = 7.13 × 10^− 11^) and vascular diseases (BH adjusted *p* value = 6.72 × 10^− 11^) as shown in Fig. 4a (Detailed information was shown in Additional file 3: Table S3). Among the significantly enriched Gene Ontology (GO) Biological Process (BP) pathways, some fibrosis-related terms stood out, such as extracellular matrix (GO:0031012) (BH adjusted *p* value = 1.55 × 10^− 15^) and regulation of blood pressure (BH adjusted *p* value = 4.10 × 10^− 7^) [33, 34]. To better interpret the results, we constructed a DEG GO term network (Fig. 4b). From this network, it would be interesting to see those genes associated with multiple terms, especially multiple categories defined by GO terms in a parent-child relationship. In our network, the gene *POSTN* was involved in 17 terms (degree = 17) in five categories. *POSTN* is critical in cardiac development and remodeling, and the expression was found to be consistent with the percentage of myocardial fibrosis [35]. Hence, genes with a high degree in this network might provide promising candidates for further investigation, such as *ACE2*, *KLF4*, *JAK2*, and *NR4A3*.

To investigate the function of the differentially expressed lncRNAs and miRNAs (hereafter, we abbreviated as DElncs and DEmiRs), we identified their potential targets. The detail information of the DElncs is provided in Additional file 4: Table S4. In total, we identified 15 potential target genes for 15 DElncs through 126 interactions. Among them, some have been already reported to be involved in cardiac diseases, such as *CORIN* and *EGLN3* [36, 37]. For the DEmiRs, 21 miRNAs have been reported to be associated with the development of HF according to the Human microRNA Disease Database (HMDD) [38]. We further used microRNA Enrichment Analysis and Annotation (miEAA) [26] to detect the significantly enriched categories. As shown in Additional file 5: Table S5, several enriched pathways were related to HF [26]. Taken together, these findings may indicate the potential roles of the DElncs and DEmiRs in the development of HF.

### Regulatory network analysis revealed novel signatures of HF

With the HF-associated coding mRNA and non-coding RNA molecules from the above analyses, we next constructed miRNA-mRNA and lncRNA-mRNA regulatory networks in order to identify novel regulators in the development of HF. Here, we only focused on the regulation between differentially expressed mRNAs, lncRNAs, and miRNAs since they are more likely to play important roles in HF.

**Fig. 5:**
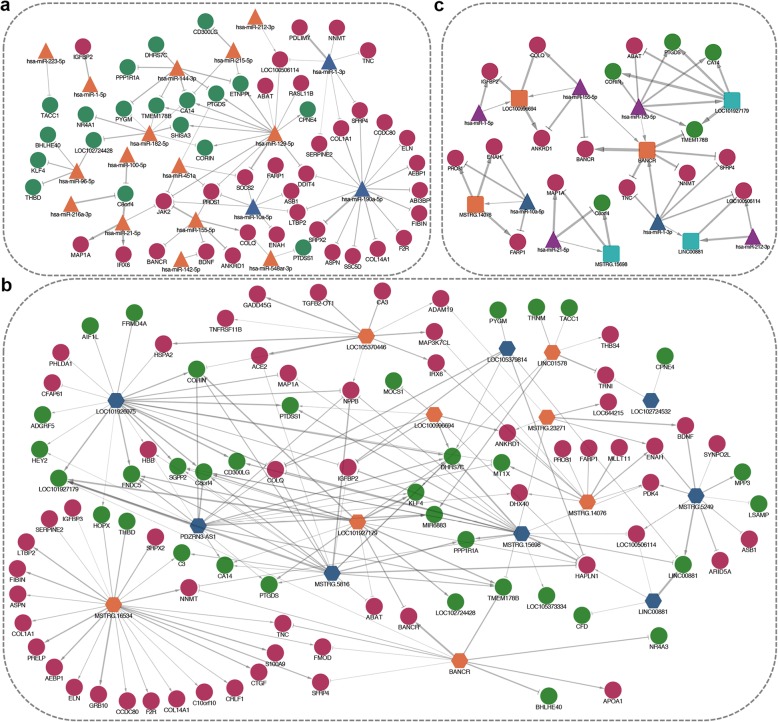
Gene expression regulatory networks in heart failure. **a** microRNA-mRNA regulatory network. **b** lncRNA-mRNA regulatory network. **c** microRNA-lncRNA-mRNA co-regulatory network. mRNAs, microRNAs, and lncRNAs are denoted by circle, triangle, and pentagon nodes, respectively. Red, orange, and purple nodes are upregulated expression and other color for downregulated expression. The edges represent differently weighted regulation. Edge width is proportional to the Spearman correlation coefficient between the linking nodes. Here, an arrow indicates activation relation while an edge ending with -| indicates repression

For DEGs and DEmiRs, the regulatory network comprised 18 DEmiRs and 63 DEGs through 86 regulatory interactions (Fig. 5a). Since ECM-associated terms are associated with fibrosis and highly significantly enriched in DEGs, we particularly examined the regulator crosstalk associated with ECM. We found that miR-1-3p, miR-155-5p, miR-190a-5p, and miR-548ar-3p associated with fibrotic genes such as *COL1A1*, *COL14A1*, and *COLQ*. Among them, miR-190a-5p (downregulated in HF samples) regulated the largest number (*n* = 16) of upregulated DEGs, most of which are involved in ECM, suggesting miR-190a-5p may be a main regulator in ECM associated with HF.

In addition to the miRNA regulatory network, the dysregulation of lncRNA expression is known to be associated with various diseases. Therefore, we constructed a lncRNA-mRNA regulatory network for HF by including DEGs and DElncs. Figure 5b shows the resultant lncRNA-mRNA network, which includes 16 lncRNAs (8 upregulated and 8 downregulated) and 92 DEGs, connected by 185 lncRNA-mRNA interactions. Among these lncRNAs, LOC101926975, LOC101927179, and MSTRG.16534 were found to interact with multiple DEGs, suggesting that they might have important regulatory roles in HF. Interestingly, the lncRNA MSTRG.16534 was associated with multiple ECM-related genes in the network, and most of these ECM genes were also regulated by miR-190a-5p. These results reinforced the important roles of ECM in the development of HF, suggesting that miR-190-5p and MSTRG.16534 might have synergistic regulatory roles in the molecular mechanism of HF.

As previous studies have reported that lncRNA could compete with miRNA and regulate miRNA-mediated target repression, we next constructed a miRNA-lncRNA-mRNA co-regulatory network. We required that miRNAs and lncRNAs have significant expression relationships based on Spearman’s correlation coefficient (*p* value < 0.05). We investigated the co-regulatory network to examine whether such co-regulations have important roles in HF. As a result, we identified several feed-forward loops (FFLs) potentially associated with HF [39]. For example, two genes *ABAT* and *PTGDS*, which were associated with multiple HF-related pathways such as regulation of blood pressure and positive regulation of muscle contraction [40], were jointly regulated by miR-129-5p and LOC101927179 as shown in Fig. 5c. The miR-129-5p was reported to be upregulated in peritoneal dialysis and was suggested to be a potential therapeutic target for the amelioration of peritoneal fibrosis in peritoneal dialysis [41].

Collectively, our regulatory network analysis identified many potential modules and molecules that might play important roles in HF development, especially fibrosis. Among them, ECM genes and their miRNA and lncRNA regulators are promising markers in HF.

### Identifying myocardial fibrosis genes based on lasso regression

**Fig. 6:**
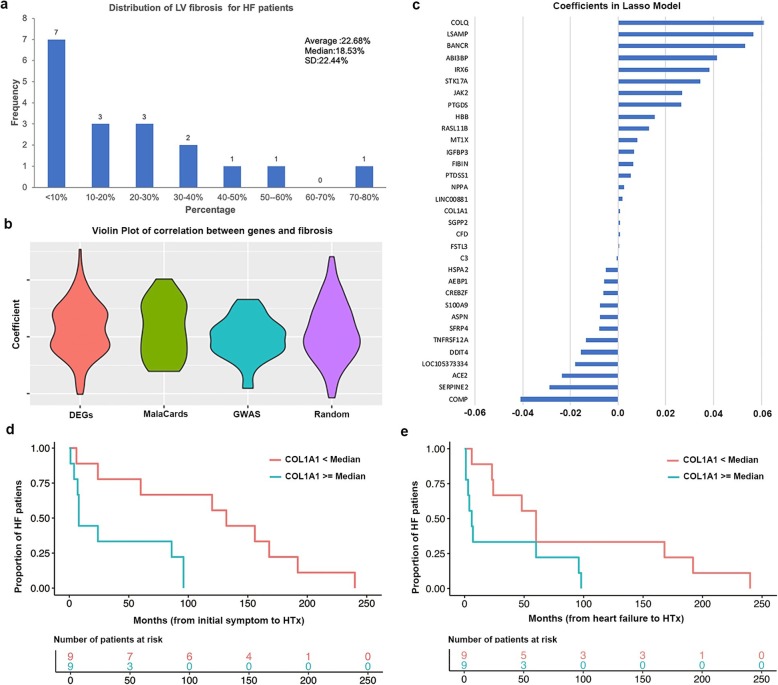
Detecting fibrosis related genes in HF. **a** Distribution of LV fibrosis percentage in the 18 HF samples. **b** Violin plot of Spearman’s correlation coefficients between fibrosis percentage and genes from three sets. **c** Selected fibrosis-related genes by coefficient based on lasso regression. **d** Two groups of HF patients as defined according to the *COL1A1* expression showed significantly different survival time (initial symptom to HTx). **e** Two groups of HF patients as defined according to the *COL1A1* expression showed significantly different time from heart failure to HTx

Based on the results mentioned above, ECM and its related genes were indicated to play important roles in the development of HF while ECM is a driver of progressive fibrosis. Here, we attempted to detect fibrosis-related genes, aiming to provide new insights into the ventricular function in HF patients. Among the 21 HF patients, 18 had available data for the percentage of fibrosis (Additional file 6: Table S6, Fig. 6a**)**. This clinical data was used for the following analysis.

We collected three sets of genes to investigate the relationship between these genes and fibrosis. These are genes previously implicated in HF, including DEGs, GWAS genes, and HF-related genes reported in literatures and available from GeneCards [42]. Figure 6b shows the distribution of Spearman’s correlation coefficient for each gene with fibrosis. We also included a set of random genes with the same size of DEGs. As shown in Fig. 6b, DEGs had the highest correlation with fibrosis compared to GWAS genes and the HF-related genes. Next, we applied the lasso regression to the DEGs to detect fibrosis-related genes.

By fitting a lasso regression model, we found 33 out of the 126 DEGs to be associated with fibrosis (non-zero coefficients), denoted as fibrosis-associated genes (Fig. 6c, Additional file 7: Table S7). Among these 33 candidate genes, we found several genes that were previously implicated in HF, such as *NPPA* (already well known for HF) and *FSTL3* (an extracellular regulator in heart [43]). Another gene is *COL1A1* which had upregulated in our study. The overexpression *COL1A1* was reported to be highly correlated with liver fibrosis [44]. Furthermore, 39.39% (13 out of 33) of the fibrosis-associated genes were included as informative genes which were defined as genes involved in pathway enrichment (Fisher’s exact test, *p* value = 1.05 × 10^− 26^). Our functional enrichment analysis of these 33 fibrosis-associated genes revealed that they were significantly enriched in extracellular space (*p* value = 3.67 × 10^− 9^), extracellular region (*p* value = 3.35 × 10^− 7^), and extracellular matrix (*p* value = 6.05 × 10^− 6^). It is well known that ECM components are subject to modulate the proliferation, migration, and activation of cardiac fibrosis. These results further supported that ECM might play a major role in the development of cardiac diseases, especially the fibrosis in HF.

### *COL1A1* as a potential fibrotic marker for HF progression

Since HF is largely a consequence of increased myocardial stiffness caused by excessive cardiac fibrosis, the percentage of the fibrosis can affect the survival of the patients. Here, we collected the time period of each patient from initial symptoms to heart transplantation (HTx) and from HF onset to HTx, respectively (Additional file 6: Table S6). In this study, the event of HTx was of our interest and we referred the survival time as the time from initial symptoms to HTx or the time from HF onset to HTx, respectively.

Based on the Pearson’s correlation coefficient between gene expression and survival information in HF samples, we identified four fibrosis-associated genes to be significantly associated with survival rate. These genes were *ASPN*, *COL1A1*, *COLQ*, and *IGFBP3*. We then applied the Kaplan-Meier test to estimate the relationship between survival data and the expression level of these genes by separating patients into two groups (one group with expression ≥ median and the other with < median). As shown in Fig. 6d, e, only one gene, *COL1A1*, showed a negative correlation with both types of survival status (*p* value = 6.1 × 10^− 3^ for initial symptoms to HTx and *p* value = 0.04 for HF onset to HTx). This is consistent with the observation that the expression of *COL1A1* was positively correlated with fibrosis and the patients with a high *COL1A1* expression needed HTx in a much shorter period than those with a low *COL1A1* expression. These results further implied that *COL1A1* represents a fibrosis signature and is associated with HF progression. Due to the relatively small number of samples, this finding warrants further validation.

### Experimental validation of COL1A1 as a potential biomarker in HF progression

**Fig. 7:**
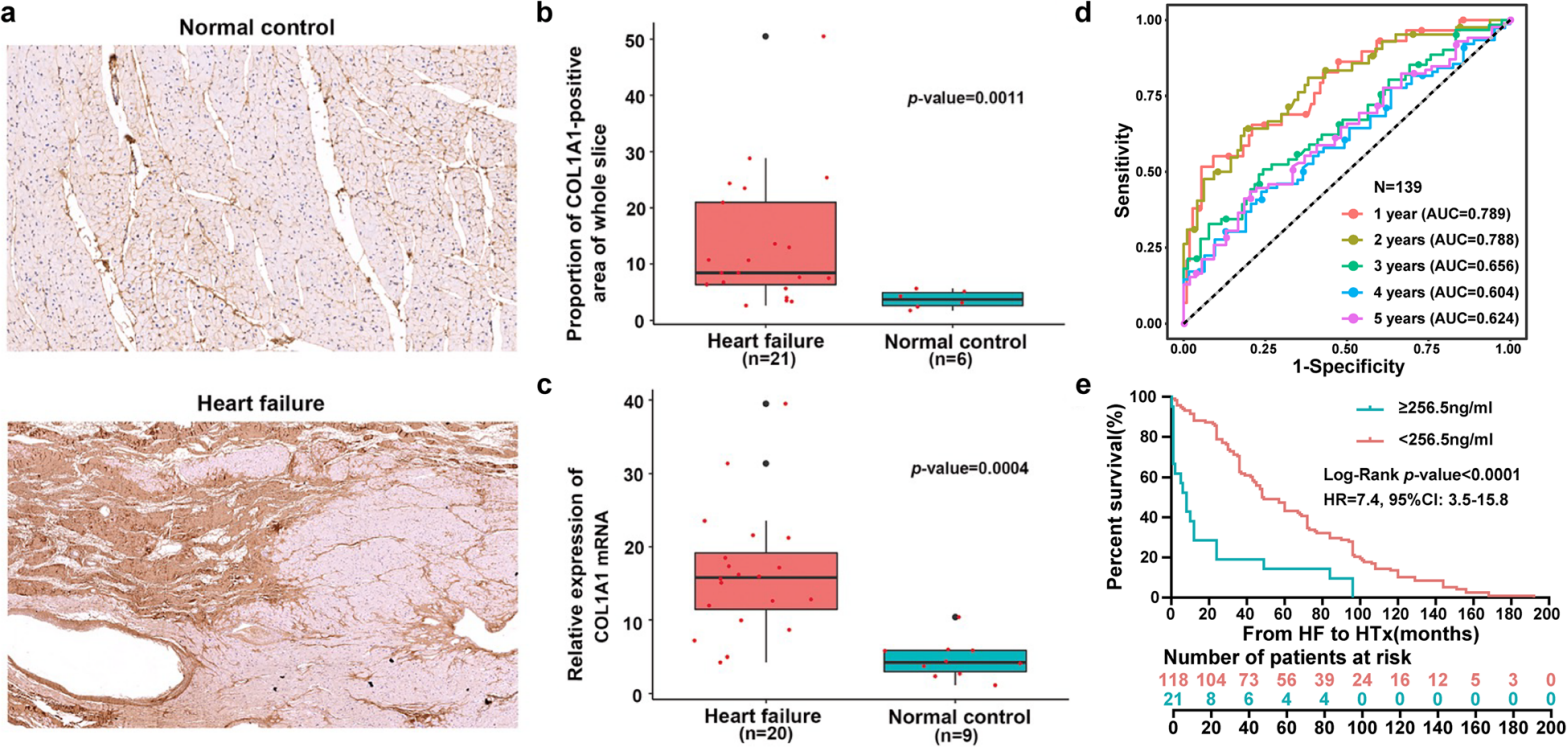
Validation of *COL1A1* gene expression by IHC and qRT-PCR and a predictor for 1-year survival from HF onset. **a** The demonstrations of IHC results of normal controls and HF patients, the upper is from a region of LV from a normal control and the under is from a HF patient. **b** IHC staining of COL1A1 shows proportion of COL1A1-positive area of whole slice in HF is higher than normal control. **c** qRT-PCR results of *COL1A1* gene expression in left ventricles of normal and HF hearts. **d** Plasma COL1A1 levels could distinguish the 1-year HTx from non-HTx when using cutoff value 256.5 ng/ml (AUC = 0.789, *p* value < 1.0 × 10^− 4^). **e** COL1A1 level ≥ 256.5 ng/ml was statistically associated with poor survival

HTx has been the most efficient treatment for end-stage HF. However, there is very limited finding regarding the biomarkers for the survival from HF onset to HTx. Our results revealed that *COL1A1* was potentially associated with fibrosis and might be a novel biomarker for HF progression. To further validate results mentioned above, we performed the immunohistochemistry (IHC) staining assay of the left ventricles in the analysis cohort. Among our 30 samples, only 21 HF samples and 6 normal samples could be further used for the IHC staining assay (Fig. 7a). Based on the quantitative analysis of COL1A1-positive area in the whole slice prepared by Imge-Pro Plus, we found that the proportion of COL1A1*-*positive area in HF was much larger than normal control (13.61 ± 2.55% vs. 3.76 ± 0.64%, *t*-test *p* value = 1.1 × 10^− 3^, Fig. 7b). To further validate the expression of *COL1A1* as a marker in HF versus normal controls, we performed the quantitative real-time PCR (qRT-PCR) assay of the *COL1A1* gene expression in an independent cohort including 20 HF samples and 9 normal controls. As shown in Fig. 7c, the relative expression of *COL1A1* showed significantly higher expression in HF samples than normal controls (4.07 ± 0.48 vs. 1.13 ± 0.22, *t*-test *p* value = 4.1 × 10^− 4^). Taken together, both IHC and qRT-PCR validation data supported that *COL1A1* was significantly upregulated in HF samples from pathological and gene transcription aspects.

Based on the previous studies, plasma biomarkers can offer great promise to further dissect the underlying disease processes, which are important in diagnosis, prognosis, and HF treatment [45, 46]. Thus, we attempted to investigate whether the COL1A1 content in plasma would be used as a biomarker for HF progression. We then examined the plasma COL1A1 content in another and a larger cohort comprising 139 HF patients, with each patient having the corresponding progression data from HF to HTx. Here, the plasma COL1A1 content was evaluated based on ELISA (see “Methods”). By calculating the Pearson’s correlation coefficient between plasma COL1A1 content and HF survival time, we found they were significantly anti-correlated with each other (Pearson’s correlation coefficient *r* = − 0.3382, *p* value < 1.0 × 10^− 4^).

The patients who progressed to HTx rapidly within 1 year is more clinically important. To examine the difference, we separated the 139 HF patients into two groups: those from HF onset to HTx within 1 year (*n* = 29) and those from HF onset to HTx by longer than 1 year (*n* = 110). Furthermore, we used this dataset as gold standard. After obtaining the plasma COL1A1 content, we can define a plasma COL1A1 content threshold for the patients being transplanted within 1 year versus longer than 1 year. In addition, the diagnostic efficiency of plasma COL1A1 expression level for survival from HF onset to HTx was also calculated. As shown in Fig. 7d, the plasma COL1A1 content could distinguish 1-year HTx group with an AUC (area under the ROC curve) score of 0.789 (cutoff value = 256.5 ng/ml, *p* value < 1.0 × 10^− 4^), in comparison with 0.656 for 3-year HTx and 0.624 for 5-year HTx. These results further suggested that plasma COL1A1 content greater than 256.5 ng/ml in plasma was statistically associated with poor survival within 1 year [hazard ratio (HR) 7.4, 95% confidence interval (CI) 3.5 to 15.8, Log-rank *p* value < 1.0 × 10^− 4^, Fig. 7e]. Taken together, our data indicated that the plasma COL1A1 content (greater than 256.5 ng/ml) might be used as a potential biomarker of HF progression, especially 1 year after onset of HF.

## Discussion

HF is a clinical syndrome caused by structural and functional defects in the myocardium resulting in impairment of ventricular filling or the ejection of blood. It has been defined as a global pandemic, and its prevalence has been increased recently, with a high rate of the associated hospitalization, morbidity, and mortality [1–3]. So far, a standardized medical treatment has been successful in the early stages of HF. However, pharmacological management has a limited role in advanced HF cases. Therefore, further studies are needed to develop novel therapeutic agents, such as regenerative and gene therapy. This requires a deep understanding of the molecular mechanisms underlying HF development and progression.

Since it is very difficult to obtain clinical patient samples with advanced HF for molecular mechanistic studies, most investigations on HF have applied animal disease models to explore the molecular mechanism of HF. In our study, we collected heart tissues from 21 HF patients and 9 healthy donors instead of using blood samples. We further performed a systematic investigation of the gene expression changes at multi-transcriptional levels and regulatory networks including mRNAs, lncRNAs, and miRNAs for HF patients. According to PCA analysis, HF and control samples were better distinguished by lncRNA expression than mRNA and miRNA expression. Although it is preliminary, this observation might motivate us to find potential lncRNA biomarkers for diagnose and prognosis of HF in future. So far, the knowledge of lncRNA in HF has been very limited. Besides, we did not collect more clinical information except sex and age of the healthy donors for the DE analysis in the present work. If we could collect more detailed information of healthy donors such as smoking and drinking, we could correct for performing DE analysis.

With the advent of anti-fibrotic pharmacologic therapies, fibrosis has become an important therapeutic target in HF. Thus, understanding the mechanisms contributing to fibrosis will help us identify therapeutic targets. In this study, we identified several genes associated with fibrosis, some of which have been reported in literature, such as *ELN* and *POSTN* [47, 48]. In addition, we identified several novel fibrosis associated genes for further validation, such as *NR4A3*, *PTGDS*, *TNC*, miR-190a, and miR-708-5p. For example, a previous study reported that PTGDS could mediate biosynthesis of PGD2 to promote cardiomyocyte survival [49]. This might imply that *PTGDS* gene identified in our study could serve as a potential therapeutic target for HF treatment. Furthermore, our network analysis revealed that microRNA: mir-129-5p and lncRNA: LOC101927179 might regulate the expression of *PTGDS* in the same network, which was associated with fibrosis (Fig. 6c) and the survival rate (Additional file 7: Table S7). Our results also consistently indicated that ECM was enriched among DEGs and fibrosis-related genes, suggesting that ECM is one major mechanism contributing to fibrosis.

The persistence of myocardial fibrosis will lead to the development of adverse changes in ventricular structure, eventually leading to the progression to HF. Although transcriptomic approaches have been applied to identify genes involved in the fibrotic process in previous studies, the specific fibrosis-related genes in HF development and further contributed to the HF progression is still not well known. With the survival rate and gene expression data in this study, we identified a fibrosis-associated gene, *COL1A1*, that was significantly associated with HF progression. By performing IHC and qRT-PCR experiments, the expression of *COL1A1* was validated to be highly upregulated in the HF samples. We were able to further investigate COL1A1 content in plasma by using another 139 HF samples. And we found that higher expression level of COL1A1 in plasma was associated with poor survival from HF to HTx (Fig. 7e). To our knowledge, this is the first report to specify fibrotic gene associated with HF progression. It is known that HF is heterogeneous with different time from HF onset to HTx ranging from 30 days to more than 5 years [50]. The plasma biomarkers might provide great promise to further dissect the underlying disease processes. Our results indicated that the plasma COL1A1 content could be a potential biomarker to distinguish the malignant process of HF within 1-year after HF diagnosis with higher diagnostic efficiency than longer survival condition (Additional file 8: Table S8). It will be helpful to assess the HF patient regarding longer survival, as well as to alleviate the overload of HF. It is important to further study the mechanistic role of *COL1A1* in fibrosis. There are some reports of the association between *COL1A1* and fibrosis in HF based on the mouse model or in other diseases such as liver cancer [51–53]. In the present work, we validated this relationship at the transcriptomic level in the human heart tissue. Instead of further investigating how *COL1A1* leads to fibrosis, we focused on the potential role of *COL1A1* in HF progression. Such finding is much needed for clinical studies in heart transplantation.

Readers should take caution of the results in our study because the sample size is still relatively small. We used 30 patients (21 HF and 9 healthy donors, all heart tissue samples) for discovery by a multi-omics approach. Our top gene (*COL1A1*) identified in the 30 discovery cohort samples was further validated by immunohistochemistry staining in the same cohort and qRT-PCR using another independent cohort (20 HF and 9 healthy donors), and an additional 139 cohort patients for evaluation of plasma COL1A1 content. This size of the heart failure cohort for potential biomarker discovery is smaller than some of the previous studies of HF [54, 55]. It is due to the fact that all the HF patients, including the 139 HF cohort, received heart transplantation, which is different from the previous studies [55]. Considering this limitation, our findings need further validation by recruiting more HF patients with related clinical data and additional functional work to illustrate specific roles of plasma COL1A1 level on HF progression in the future. Furthermore, most HF patients included in the study for multi-transcriptomic analysis were young (mean age 34.6), and much younger than the general patient population. Our preliminary analysis did not find any significant difference by checking family history and etiology of these HF patients. Since most HF patients are older than our samples, the results in this study might include some unique features of this specific population and further investigation is warranted in future. Moreover, we found that there were two HFpEF samples in our study cohort. In future, when there is larger cohort and more comprehensive data available, we will extend the study of mechanism of early-onset and late-onset HF as well as the different subtypes of HF.

## Conclusions

We performed a systematic investigation of the gene expression at multi-transcriptional levels and then explored a co-regulatory network using differentially expressed mRNAs, miRNAs, and lncRNAs. Our network analysis not only provided a high-level view of the functional changes but also pinpointed several critical regulators. By examining the relationship between fibrosis percentage and gene expression, we identified several genes and their regulatory networks that might be related to fibrosis. Furthermore, the fibrosis associated gene *COL1A1* was found to be associated progression of HF. COL1A1 content in plasma could be used as a potential biomarker for HF progression, especially for predicting the 1-year survival from HF onset to HTx.

## Supplementary information

**Additional file 1:** **Table S1.** Summary of the basic information of HF patients and normal controls included in the study.

**Additional file 2:** **Table S2.** The RNA-seq statistics and alignment information for each sample.

**Additional file 3:** **Table S3.** Functional enrichment analysis of differentially expressed mRNAs by online tool WebGestalt.

**Additional file 4:** **Table S4.** The detailed information of differentially expressed lncRNAs.

**Additional file 5:** **Table S5.** Functional enrichment analysis of differentially expressed microRNAs by using tool miEAA.

**Additional file 6:** **Table S6.** Fibrosis percentage and survival data for each heart failure sample.

**Additional file 7:** **Table S7.** Fibrosis related genes identified by lasso regression analysis.

**Additional file 8:** **Table S8.** Evaluation of survival (years) by using plasma COL1A1 level of HF patients.

## Data Availability

The datasets used and/or analyzed during the current study are available at the Gene Expression Omnibus (GEO) under accession code GSE135055.

## Abbreviations

AUC: Area under the ROC curve
BH: Benjamini and Hochberg
BP: Biological Process
cDNAs: Complementary DNAs
CI: Confidence interval
CMR: Cardiac magnetic resonance
DEGs: Differentially expressed genes
ECM: Extracellular matrix
ELISA: Enzyme-linked immunosorbent assay
FC: Fold change
FFLs: Feed-forward loops
FPKM: Fragments per kilobase of transcript per million mapped reads
GO: Gene ontology
HF: Heart failure
HMDD: Human microRNA Disease Database
HR: Hazard ratio
HFpEF: Heart failure with preserved ejection fraction
HFrEF: Heart failure with reduced ejection fraction
HTx: Heart transplantation
IHC: Immunohistochemistry
lncRNA: Long non-coding RNA
LV: Left ventricular
LVEF: Left ventricular ejection fraction
miEAA: MicroRNA Enrichment Analysis and Annotation
miRNA: MicroRNA
ORA: Over Representation Analysis
PCA: Principal component analysis
qRT-PCR: Quantitative real-time PCR
SD: Standard deviation
